# Pulpitis as a microbial disease: single-cell insights into host responses and diagnostic biomarkers for vital pulp therapy

**DOI:** 10.1080/20002297.2026.2622207

**Published:** 2026-02-17

**Authors:** Tiansong Xu, Daishan Yang, Liqi Zhang, Murong Li, Xiuhua Li, Chenggang Duan, Yoo Cheung, Wen Zhang, Zhijian Zhang, Lin Yue, Xiaoying Zou, Feng Chen

**Affiliations:** a Fifth Clinical Division, Peking University School and Hospital of Stomatology & National Center for Stomatology & National Clinical Research Center for Oral Diseases & National Engineering Research Center of Oral Biomaterials and Digital Medical Devices, Beijing, People's Republic of China; b Central Laboratory, Peking University School and Hospital of Stomatology & National Center for Stomatology & National Clinical Research Center for Oral Diseases & National Engineering Research Center of Oral Biomaterials and Digital Medical Devices, Beijing, People's Republic of China; c Lingchuan Stomatology Inc., Beijing, People's Republic of China; d Department of Cariology and Endodontology, Peking University School and Hospital of Stomatology & National Center for Stomatology & National Clinical Research Center for Oral Diseases & National Engineering Research Center of Oral Biomaterials and Digital Medical Devices, Beijing, People's Republic of China; e Department of Stomatology, Peking University International Hospital, Beijing, People's Republic of China; f Department of Respiratory and Critical Care Medicine, The Second Medical Center and National Clinical Research Center for Geriatric Disease, Chinese PLA General Hospital, Beijing, People's Republic of China; g Center of Stomatology, Peking University Hospital, Beijing, People's Republic of China

**Keywords:** Pulpitis, oral microbiome, dental pulp stem cells (DPSCs), single-cell RNA sequencing, biomarkers, vital pulp therapy

## Abstract

**Background:**

Pulpitis is a common dental disease driven by complex microbial infections, yet its microbial origins, diversity, and pathogenic mechanisms remain incompletely understood. A major clinical challenge is the absence of objective biological criteria to assess the severity and reversibility of pulpal inflammation, which is essential for decision-making in vital pulp therapy (VPT).

**Objective:**

This review aims to synthesize current evidence on the microbial landscape of pulpitis and to explore microbial- and host-derived biomarkers that may enable objective assessment of inflammation severity and support precision VPT.

**Design:**

We comprehensively reviewed microorganisms implicated in pulpitis and their distinct virulence mechanisms underlying inflammatory responses and tissue damage. Particular emphasis was placed on host responses of dental pulp stem cells (DPSCs) to different microbial infections. Biomarker candidates reported across multiple studies were summarized, and single-cell transcriptomic evidence was integrated to validate microbe-specific DPSC responses.

**Results:**

Distinct microorganisms associated with pulpitis exhibit heterogeneous virulence strategies, inducing diverse inflammatory and degenerative processes within the dental pulp. DPSCs display infection-specific transcriptional responses, revealing molecular signatures linked to inflammation severity and tissue repair potential. Emerging biomarkers derived from both microbial factors and host responses show consistency across studies, with single-cell analyses providing high-resolution validation of these microbe-specific patterns.

**Conclusions:**

Microbial- and host-derived biomarkers hold significant translational potential for stratifying pulpitis severity, informing VPT decision-making, and predicting treatment prognosis. Integrating microbial characterization with host response profiling may advance objective diagnosis and personalized management of pulpitis.

## Key messages

- The histopathology of pulpitis is fundamentally driven by the interaction between pathogens and host cells, particularly dental pulp stem cells (DPSCs), which respond by releasing a spectrum of enzymes and cytokines.
- Single-cell transcriptomic analyses further reveal that this interaction induces both homogeneous and heterogeneous responses across distinct DPSC subclusters.
- Our findings deepen our understanding of pulpitis pathogenesis and pave the way for improved therapeutic strategies for primary periodontal disease with secondary endodontic involvement.

## Introduction

The human oral microbiota is highly diverse and exhibits a complex ecological structure, consisting of bacteria, microeukaryotes, archaea and viruses [1–4]. These microorganisms collectively create intricate and highly organised oral microenvironments, which can contribute to the development of oral and related diseases, including dental caries, periodontitis, pulpitis and apical periodontitis [2,4–6].

Among these, dental caries, a prevalent chronic degenerative condition affecting almost 2.0 billion throughout the world, poses significant health concerns [7]. This disease, driven by microbial infection, disproportionately affects individuals in developing countries, where its prevalence is notably elevated. If left untreated, microbes can penetrate the pulp through damaged tooth tissue, leading to pulpitis and periapical inflammation, which may ultimately result in tooth loss, abscess formation or even systemic infections in severe cases. While conventional root canal therapy remains the gold standard for irreversible pulpitis, it inevitably results in the loss of pulp vitality and its biological functions.

In recent years, vital pulp therapy (VPT) has emerged as a biologically favourable and less invasive alternative, aiming to preserve the vitality and function of the inflamed pulp tissue [8,9], is considered a promising direction for future endodontic therapy. However, the widespread clinical adoption of VPT is still limited, primarily due to the lack of objective and reliable diagnostic criteria to evaluate the extent and severity of pulpal inflammation. Currently, treatment decisions largely depend on subjective symptoms, intraoperative bleeding patterns, microbial presence and radiographic interpretation, which may not accurately reflect the underlying biological state of the pulp.

Although the microbial aetiology of pulpitis has been well established, emerging evidence highlights that the microbial composition associated with pulp inflammation is more diverse and context-dependent than previously thought. Microorganisms associated with pulpitis may originate from different routes, including carious lesions, periodontal pockets or haematogenous spread, particularly under conditions such as systemic bacteraemia or trauma [10–12]. These differences are reflected in the host immune responses, particularly those mediated by dental pulp stem cells (DPSCs), which play critical roles in inflammation regulation, tissue repair and regeneration [13–15].

We previously hypothesised that the heterogeneity of microbial origins may induce distinct cellular responses in DPSCs, potentially correlating with different infection mechanisms, as well as the stages or severities of pulpitis. To test this, we investigated the single-cell transcriptomic profiles of DPSCs in response to *Streptococcus mutans* (*S. mutans*), *Enterococcus faecalis* (*E. faecalis*), *Candida albicans* (*C. albicans*), *Porphyromonas gingivalis* (*P. gingivalis*) and *Capnocytophaga periodontitidis* (*C. periodontitidis*). By characterising these microbe-induced host responses at single-cell resolution, we aimed to identify molecular signatures and pulp states under different infection mechanisms, which might serve as biomarkers for inflammation grading and ultimately assist in clinical decision-making for VPT.

This present review focuses on pulpitis and inflammatory states of the dental pulp, with an emphasis on biological processes relevant to diagnosis and vital pulp therapy. We first summarise the current understanding of the microbial origins and virulence characteristics of pulpitis-related pathogens. We then explore recent single-cell transcriptomic insights into how DPSCs respond to infections from distinct microbial sources. Finally, we discuss the potential of leveraging host response patterns to identify diagnostic biomarkers that can help stratify pulp inflammation and guide personalised treatment strategies in the context of vital pulp therapy.

## Pathology and cause of pulpitis

### Aetiological factors

**Figure 1.**
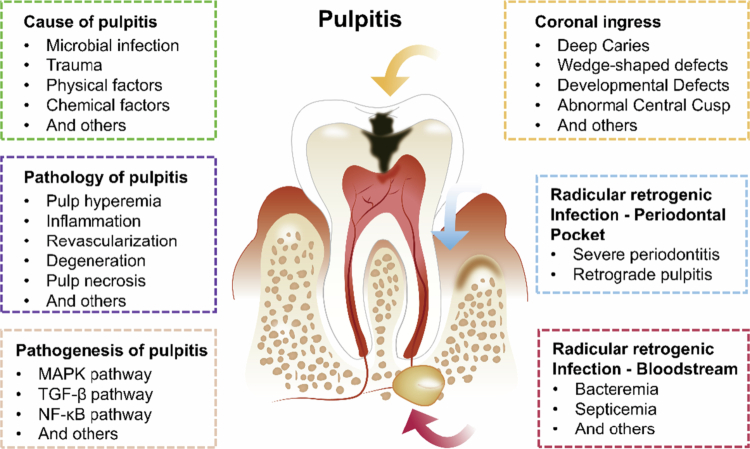
Schematic illustration of the cause, pathology and pathogenesis of dental pulpitis caused by coronal ingress and radicular retrogenic infection.

Pulp inflammation is a dynamic and complex process involving neural, vascular and immune responses, which might be triggered by many factors, including triggered by microbial infection, chemical or mechanical irritants and trauma (Figure 1).

#### Microbial infection

Microbial infection is the most frequent cause of pulp diseases. Among these, caries-induced pathogen are the primary contributors to pulpal inflammation and infection. The progression of pulpal injury is a complex, dynamic process influenced by both the invading microorganisms and the response of host, such as inflammation and immune defences [12].

Studies using experimental pulpitis models have shown that bacterial antigens and metabolic by-products was able to diffuse through dentinal tubules, triggering immune responses in the dental pulp.

Pulp inflammation primarily arises from the interaction between anaerobic bacteria, such as *Porphyromonas gingivalis*, *Prevotella intermedia*, *Fusobacterium nucleatum* and *Treponema denticola*, along with other microbes [16,17]. However, no specific microbial species have been directly linked to particular clinical symptoms [17]. In cases with inflamed pulp, microbial determination indicated that species of the microorganisms were consistent with clinical diagnosis and were associated with the severity of pulpitis [18]. It was necessary to identify suspected endodontic pathogens in the aetiology of each form of pulpal diseases to determine the best therapeutic measures [17].

#### Other factors

Aside from microbial infection, various traumatic, physical and chemical factors can also irritate the pulp and trigger inflammation. Both acute and chronic trauma may compromise pulpal health: sudden injuries such as crown fractures, luxations or root fractures can disrupt the blood supply and directly expose the pulp to microbial contamination, leading to pulpitis or necrosis.

Long-term traumatic forces, such as occlusal trauma, attrition and erosion, may gradually impair pulp circulation and predispose to chronic inflammation [19,20]. Short-term stimuli, including cavity preparation and restorations, may adversely impact the pulp, leading to acute inflammation. Enamel and dentin, among the hardest tissues in the body, can transfer the heat generated during cavity preparation, potentially inducing pulp inflammation [21,22]. Approximately 9% of patients who receive large restorations eventually develop pulpal disease. The risk is particularly high among older patients receiving extensive (4-surface) amalgam restorations [23].

Chemical factors likewise play a role. Disinfectants, such as phenols or silver nitrate, as well as acidic components in restorative materials and bonding agents, could irritate the pulp if dentin thickness is insufficient.

In addition, chronic irritation from leaky or deteriorated restorations, erosive agents or dietary acids may sustain low-grade inflammation over time. Beyond local factors, systemic conditions such as sickle cell anaemia, AIDS and HIV infection have been associated with pulp pathology, while barometric pressure changes during flying or diving may precipitate pulp barotrauma and intrapulpal haemorrhage.

### Histopathology of pulpitis

Achieving successful therapeutic outcomes in vital pulp therapies primarily relies on accurately assessing pulp status. However, diagnosing pulpal pathology is often highly complex.

Inflammation is primary reaction of the pulpitis, similar to other inflammation in other tissue. A key difference from other tissues is that the pulp is encased within rigid, highly mineralised dentin walls, restricting the lymphatic system's ability to drain extravasated fluid, inflammatory mediators and leucocytes. This anatomical limitation results in harmful effects, including an accumulation of extravasated fluid in the pulp tissue, oedema formation, increased interstitial pressure, compression of blood vessels, intrapulpal blood stasis and ultimately tissue hypoxia and necrosis [24,25]. Then, pulp has favourable and unfavourable responses. On the one hand, the root canal system aims to recover and return to normal situation, including tertiary dentine formation, pulp revascularization, fibrosis and canal calcification; on the other hand, pulp can defend against adverse stimulation and emerge pulp necrobiosis, internal root resorption surface, inflammatory and/or replacement [20].

Different classifications of pulpitis have distinct pathological features, mainly including reversible pulpitis and irreversible pulpitis. In the reversible pulpitis stage, 13.3% of the samples exhibited severe hyperaemia, and only some slight changes in an odontoblastic layer without inflammatory cells are typically observed in a pulp hyperaemic condition [26]. Irreversible pulpitis is marked by the presence of bacteria or their by-products within dental tubules and pulp tissue near deep caries. It is also primarily indicated by neutrophils in the tissue beneath the lesion, which suggests neutrophil-chemotactic activity. Lysosomal enzymes released by neutrophils cause extensive tissue damage and lead to pus formation [26–28]. Irreversible pulpitis included two basic types: acute and chronic pulpitis. The morphological characteristics of acute inflammation involve two key processes: vascular changes and cellular events, while chronic inflammation was associated with infiltration of tissue with macrophages, lymphocytes and plasma cells, significant tissue damage along with ongoing repair processes characterised by angiogenesis and fibrosis [29]. Compared acute and chronic pulpitis, dilated blood vessels were found in 56.5% of patients with acute pulpitis and 15.2% of those with chronic pulpitis. Neutrophilic leucocytes appeared in 43.5% of acute pulpitis cases and 69.7% of chronic pulpitis cases. Lymphocytes were present in 17.4% of acute pulpitis samples but absent in chronic pulpitis samples [30].

In terms of immune responses, it is present in the healthy pulp to restrain the initial spread of pathogens. In the inflamed pulp, the immune responses prepare the conditions for necrosis or regeneration to maintain pulp tissue homoeostasis [31]. Its defence mechanisms are divided into innate and adaptive immune responses. These immune responses are associated with the pathology of pulpitis and contribute to varying prognoses [32]. These immune responses involve the coordinated actions of multiple cell subsets, including neutrophils, macrophages, lymphocytes, dendritic cells and odontoblasts, which collectively mediate pathogen recognition, inflammatory amplification and tissue remodelling.

In addition, tertiary lymphoid structure (TLS) was found to emerge in dental pulp with pulpitis, which is consistent with the high expression of CC chemokine ligand 3 (CCL3), which may be a key driver of TLS formation. Dental pulp can generate a directional immune response to bacterial infection based on TLS [33]. Within this multicellular immune microenvironment, resident stromal cells such as dental pulp stem cells (DPSCs) interact dynamically with immune cells, contributing to inflammatory regulation and subsequent repair processes, thereby providing a biological link to later discussions on DPSC-centred host responses and biomarker discovery.

### Molecular pathogenesis of pulpitis

Pulpitis is caused by the activation of the biological defence mechanism of the dental pulp against cariogenic bacteria and other pathogenic factors. The activation of multiple signalling pathways and related genes participated in this process.

The mitogen-activated protein kinase (MAPK) signalling cascade involves up to six tiers, enhancing both the amplification and specificity of transmitted signals. This cascade ultimately activates various regulatory molecules in the cytoplasm and nucleus, initiating cellular processes such as proliferation, differentiation and development [34,35]. Similarly, the activation of the MAPK signalling pathway has been confirmed by multiple studies to emerge during the process of pulpitis. Botero et al. was confirmed that LPS-induced VEGF was related to phosphorylation of protein kinase C (PKC zeta) and extracellular signal-regulator kinase (ERK1/2). These effects are dependent upon MAPK activation in DPSC and human dental pulp fibroblasts (HDPF) [36]. And NOD-1 might be involved in pulp inflammation through chemokine production via MAPK signalling pathways [37]. Xiong et al. was reported that IL-17 might participate in pulp tissue inflammation through chemokine production and NF-κB and MAPKs signalling pathways [38].

The transforming growth factor-beta (TGF-*β*) superfamily has been implicated in many aspects of the regulation of cell growth, differentiation and function. TGF-beta 1 was found in the odontoblastic–subodontoblastic layer of irreversible pulpitis specimens, indicating a role for TGF-beta 1 in the dentinal repair processes after pulp inflammation [39]. TGF-*β* was reported to induce collagen deposit around newly recruited polymorphonuclear cells to keep from microbial diffusing [40]. TGF-beta1 could inhibit the expression of TLR2 and TLR4 and attenuate odontoblast responses, associated with poor response to *Streptococcus mutans*, *Enterococcus faecalis* and *Lactobacillus casei* [41].

The nuclear factor κB (NF-κB) family, which is composed of various combinations of Rel proteins, is widely implicated in inflammatory diseases and even cancer [42–44]. In pulpitis, NF-κB signalling becomes abnormally activated, leading to alterations in related cellular differentiation. NF-κB signalling regulates the expression of pro-inflammatory molecules and affects the differentiation of DPCs and HDPFs, aiding in the management of pulp inflammation. This regulatory role helps prevent excessive inflammation in the dental pulp and supports the viability of pulp tissues, particularly during the early stages of inflammation [42].

In the development process of pulpitis, the signalling pathways involved above are a comprehensive process. Conditioned medium from rDFSCs (rDFSC-CM) downregulated the ERK1/2 and NF-κB signalling pathways in lipopolysaccharide (LPS)-induced inflammatory rat dental pulp cells (rDPCs), which resulted in suppression of the expression of IL-1β, IL-6 and TNF-*α* and promotion of the expression of IL-4 and TGF-*β*, and leaded to the attenuation of rDPC inflammation [45]. In our study, we also found MAPK, TGF-*β* and NF-κB signalling pathways were activated in DPSCs infected by *Streptococcus mutans* and *Candida albicans* [15].

Additionally, epigenetics plays a crucial role in pulpitis, impacting processes like inflammation and endodontic regeneration [46,47]. Long noncoding RNAs (lncRNAs) was reported to modulate numerous pathological and biological processes in the inflammation and regeneration of pulpitis. TFAP2A-AS1 inhibited odontogenic differentiation while promoting inflammation in pulp cells. It reduced levels of dentine sialophosphoprotein, dentin matrix protein-1 and ALP activity. The knockdown of TFAP2A-AS1 alleviated LPS-induced inflammation and enhanced cell proliferation in hDPSCs [48]. MEG3 was significantly upregulated in both inflamed pulp and LPS-treated hDPCs. The downregulation of MEG3 inhibited the secretion of TNF-*α*, IL-1β and IL-6 in LPS-treated hDPCs via the p38/MAPK pathway, while the knockdown of MEG3 promoted the odontogenic differentiation of hDPCs by regulating the Wnt/β-catenin pathway [49]. LPS-induced cell injury in pulpitis can be promoted by DUXAP8 through the miR-18b-5p/HIF3A axis [50].

In addition to the above pathways, there are also some pathways that have been studied in the pathogenesis of pulpitis. Thermosensitive transient receptor potential (TRP) ion channels expressed in the dental pulp may be key transducers of inflammation and nociception, as evidence thatTRPV3, TRPM2 and TRPM3 expressions were much lower in inflammatory conditions of human dental pulp cells (hDPCs) [51]. Growth differentiation factor 11 (GDF11) could promote SIRT3/FOXO3-mediated mitophagy to accelerate osteogenic/odontogenic differentiation in DPSCs [52]. Additionally, reactive oxygen molecules (ROS) level increased in inflamed dental pulp tissue [53].

Collectively, these signalling pathways reflect the underlying biological severity and regenerative potential of pulpal inflammation, providing a mechanistic basis for clinical decision-making in the management of pulpitis.

## Treatment of pulpitis and diagnostic challenges

### Treatment strategies

**Figure 2.**
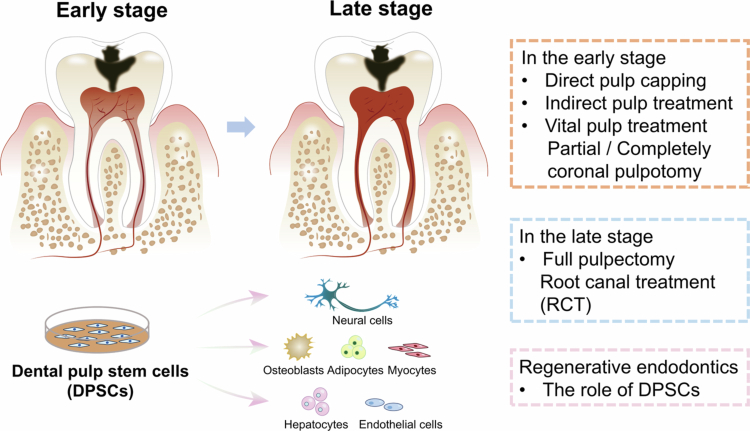
Summary of the treatment of pulpitis and regenerative endodontics based on dental pulp stem cells (DPSCs).

The principle of treating pulp disease is to preserve the vital pulp or at least retain the affected tooth to maintain chewing and other functions. According to W J Wolters *et al.*, most cases of adult pulpitis can be treated with direct pulp capping, indirect pulp treatment (IPT) or partly/completely coronal pulpotomy to preserve the vital pulp. However, if the bleeding persists after rinsing with 2 mL of 2% NaOCl when operating partly/completely coronal pulpotomy, then the vital pulp cannot be preserved and a full pulpectomy needs to be performed [54]. Besides, the affected tooth should be maintained whenever possible (Figure 2).

### Diagnostic limitations and biomarker-based strategies

Current indications for VPT rely mainly on clinical symptoms, which are often unreliable. Clinical evaluation alone cannot distinguish reversible from irreversible pulpitis, whereas biomarker quantification may provide greater accuracy [55]. Recent studies highlight that molecular alterations – such as changes in cytokines, chemokines and matrix metalloproteinases – precede histological damage and thus hold promise for early, non-invasive diagnosis [56]. Molecular-based strategies are therefore emerging as valuable tools for differentiating pulp status and guiding treatment. In the following section, we summarise key studies and potential biomarkers reported in the literature.

#### Evidence from systematic reviews

The diagnostic accuracy of vital pulp therapy (VPT) depends on distinguishing healthy from inflamed pulp. Rechenberg et al. reviewed 57 studies and identified 64 biomarkers (71.9%) with significant differences between irreversible pulpitis and healthy pulp, including IL-8, CXCL-10, MIP family, MCP family, RANTES, Eotaxin and IP10. These TLR-induced chemotactic molecules can be detected not only in pulp tissue but also in gingival crevicular and dentinal fluid, providing non-invasive diagnostic potential [27]. Zanini et al. reviewed 32 studies and reported increased expression of IL-8, MMP-9, TNF-*α* and RAGE, although evidence for RAGE remains limited [55]. A mini-review further emphasised MMP-8, IL-8, Substance *P* and Neurokinin A as promising biomarkers in gingival crevicular fluid and dentinal fluid of symptomatic irreversible pulpitis (SIP) patients [57].

#### Insights from bioinformatics and high-throughput studies

Large-scale transcriptomic datasets have expanded candidate biomarkers. Chen et al. integrated GEO datasets and identified 470 DEGs enriched in inflammatory pathways (cytokine–cytokine receptor interaction, chemokine, NF-κB), highlighting hub genes IL-6, CXCL8, MMP9, ICAM1 and others [58]. Li and Sun identified 87 chromatin remodelling–related DEGs and validated TNF, STAT3, MYC, ACTB and MAPK8 as diagnostic markers using qRT-PCR. They also constructed ceRNA and drug–gene networks, proposing novel regulatory axes and therapeutic targets [59]. Xin et al. identified 843 DEGs from GEO, extracting CXCL10, CXCL1, CCL5 and CXCR4 as hub genes through PPI network analysis [60].

#### Biomarkers for differentiating disease stages

Beyond general inflammation, biomarkers that discriminate reversible from irreversible pulpitis are most clinically valuable. Loo et al. analysed pulp blood and identified IL-1β, TGFα and FGF-2 as discriminators of disease stage, while IL-1α, IL-13, IL-17A, IL-22 and MMP2 differentiated symptomatic irreversible pulpitis from asymptomatic irreversible pulpitis [61]. Al-Natour et al. validated IL-8, IL-6, MMP9 and novel markers (CCL21, MT1H, AQP9) and identified fluvastatin as a therapeutic candidate based on biomarker modulation [62]. Brizuela et al. showed IL-1α, VEGF-*α* and FGF acid to be significantly elevated in irreversible cases, achieving an AUC-ROC of 0.92 when combined with IL-6 and TIMP-1 [63].

#### Systematic screening and clinical translation

Donnermeyer et al. systematically reviewed 28 studies and reported consistent upregulation of proteins and cytokines including IL-1β, IL-2, IL-6, IL-8, osteocalcin, PGE₂ and eNOS in inflamed pulp [13]. PGE₂ expression and its correlation with osteocalcin were proposed as indicators distinguishing pulpitis stages. Nonetheless, translation into clinical practice requires standardised thresholds, validation of mediator interactions and rapid chairside diagnostic assays.

Collectively, these studies demonstrate significant progress in identifying molecular signatures of pulpitis through reviews, bioinformatics and clinical studies. However, reliable biomarkers for grading disease severity remain elusive. Future work should validate biomarker panels in larger cohorts and integrate them into non-invasive diagnostic tools to improve decision-making in VPT. Given these diagnostic limitations, increasing attention has shifted toward biological strategies that not only identify disease but also aim to restore pulp vitality, thereby laying the foundation for regenerative endodontics.

### Dental pulp stem cells and regenerative endodontics

Based on the limitations of conventional endodontic treatments, the concept of regenerative endodontics was proposed in the 1960s, primarily to address the management of teeth with pulp necrosis [64]. The central goal of regenerative endodontics is to restore pulp vitality and function in necrotic teeth, ultimately enabling regeneration of the pulp-dentin complex [65]. Current regenerative endodontic procedures (REPs), including root canal revascularization, cell homing strategies and stem cell-based approaches, are therefore designed for non-vital teeth rather than inflamed but vital pulp tissue. Accordingly, these procedures fall outside the direct therapeutic scope of pulpitis and vital pulp therapy [8,66].

Recently, stem cell transplantation was reported to successfully regenerate pulp tissue in clinical practice. The stem cell transplantation strategy offers promising potential for pulp regeneration by introducing exogenous stem cells into an empty root canal, with the goal of regenerating a complete pulp-dentin complex. Stem cells, biomaterials and conducive microenvironments were the critical factors to the success of stem cell transplantation [67–69]. Dental-derived mesenchymal stem cells (MSCs) were applied in these strategies owing to their ready availability and inherent regenerative potential.

#### Biological properties of DPSCs

In dental pulp, several cell types of dental pulp have been identified, including odontoblasts, fibroblasts, dental pulp stem cells, endothelial cells and T cells [70,71]. Among them, dental pulp stem cell (DPSC) is the important type of dental pulp cells, which could repair human tissues, secrete inflammatory cytokines to maintain homoeostasis and have immense potential for tissue regeneration [68,72,73]. Dental pulp stem cells could be categorised into two types based on their origin: dental pulp stem cells (DPSCs) and stem cells from human exfoliated deciduous teeth (SHED). In 2000, Gronthos et al. confirmed the presence of DPSCs in permanent teeth. Later, SHED was found within the pulp chambers of exfoliated deciduous teeth in 2003. Both DPSCs and SHED are types of MSCs with clonal formation, high proliferative capacity and self-renewal potential [74,75].

Compared to other stem cells, DPSCs have several general advantages. First, DPSCs can be obtained from the pulp of extracted teeth, making them easily accessible; they are subject to less ethical controversy than embryonic stem cells or induced pluripotent stem cells; in autologous transplantation, DPSCs effectively avoid immune rejection and the need for immunosuppressants; and they pose a lower risk of tumour formation after transplantation. Therefore, establishing a stem cell bank for dental pulp stem cells is more convenient, safe and feasible. Consequently, DPSCs are considered one of the most promising stem cell sources for clinical and tissue engineering applications [76]. Additionally, due to their unique biological characteristics compared to other MSCs, DPSCs have been extensively studied for applications in tissue regeneration, especially in the regeneration and homoeostasis regulation of pulp tissue [77,78].

In terms of pulpitis treatment, Ustiashvili et al. revealed that the number of pulp stromal cells decreased and cell proliferation activity reduced during the acute pulpitis. However, during the chronic transition phase, stem cells migrated to the periphery of the pulp, leading to the maturation of B cells, which in turn increases the number of active odontoblasts capable of producing dentin, initiating reparative dentin formation [79]. Additionally, Iohara et al. transplanted CD105^+^ DPSCs into the root canal of dogs after pulpectomy with stromal cell-derived factor-1. By day 14, the root canal was successfully filled with regenerated pulp tissue, including nerves and blood vessels, and new dentin had formed along the dentin walls [80].

#### Preclinical and clinical studies on dental pulp regeneration of DPSCs

Several studies were performed transplantation of human clinical-grade DPSCs to evaluate the utility of the stem cell therapy in a pilot clinical study.

In 2017, Nakashima et al. from Japan reported a preliminary clinical study on pulp regeneration using autologous DPSCs transplantation. The study recruited five patients with irreversible pulpitis and conducted a 24-week follow-up. The results showed strong positive responses in pulp vitality tests at the 4-week follow-up. After 24 weeks, the magnetic resonance imaging (MRI) signal intensity of the regenerated tissue within the root canal was similar to the normal dental pulp in untreated control groups [81]. Subsequently, a randomised controlled clinical trial in China, which involved the transplantation of autologous SHED aggregates, further confirmed the reliability of SHED for pulp regeneration. The study recruited 40 patients with pulp necrosis following traumatic dental injuries. After 12 months, laser Doppler flowmetry and pulp vitality testing indicated regeneration of vascular and sensory nerve pulp tissues. Three-dimensional full pulp tissue regeneration was confirmed, including the formation of odontoblast-like cell layers, connective tissue, blood vessels and nerves [77,82]. Then, Nakashima et al. also used DPSCs to reconstruct the pulp in multi-rooted molars for two patients. After 4 weeks of follow-up, the pulp vitality test showed a positive response. After 24 weeks, the MRI signal intensity of the regenerated tissue was similar to that of the normal pulp in adjacent teeth [83].

These preliminary clinical studies demonstrated that DPSCs are safe and effective for complete pulp regeneration in human pulp. However, infections in inaccessible areas within the root canal system and endodontic lesions can cause resistance and obstruction, ultimately hindering the success of treatment [84,85]. It is crucial for the success of dental pulp regeneration to understanding the impact of microbial infections on DPSCs.

Given that pulpitis is fundamentally a microbial-driven disease, the nature and origin of the infecting microorganisms may critically influence the biological state of the pulp. Microbes can invade through different routes – such as carious lesions, periodontal pockets or haematogenous spread – each presenting distinct virulence factors, ecological niches and infection dynamics. These differences in microbial origin not only shape the progression of inflammation but also drive distinct host immune and repair responses within the dental pulp. Based on this, we next describe the major microbial species implicated in pulpal infections and their associated host response mechanisms, with the aim of identifying potential pathogen-specific biomarkers and underlying pathways that could aid in assessing the inflammatory status of the pulp.

## Principal pulpitis-associated microbes

**Figure 3.**
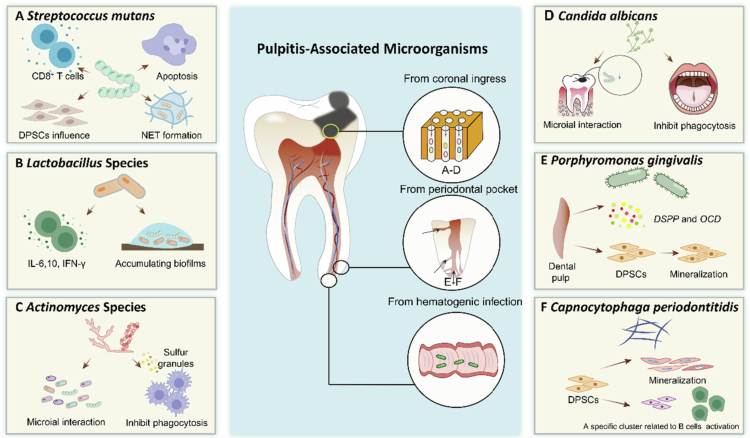
Illustration of microorganisms associated with pulpitis infection and their pathogenic mechanisms, originating from coronal pathways (A–C), periodontal pockets (D–E) and haematogenous spread (F), including *Streptococcus mutans* (A), *Lactobacillus* species (B), *Actinomyces* species (C), *Candida albicans* (D), *Porphyromonas gingivalis* (E) and *Capnocytophaga periodontitidis* (F).

Since the choice of pulpitis treatment, such as vital pulp treatment or root canal treatment, depends on the extent of the pathogen infection [86]. It was important to make sense of classical types of pathogens and related inflammatory signalling pathways [13]. Microbial was changing along the progression of pulpitis dynamically. Taxa associated with the status of caries-induced pulpitis were widely identified [18]. We concluded profiling of the root canal microbiome, the typical taxa from two sources, including coronal ingress and radicular retrogenic infection, respectively (Figure 3).

### Microbial profiling in endodontic infections

Conventional methods have provided valuable insights into the microbial composition and diversity of various endodontic infections, though they are limited by inherent constraints. Culture techniques fail to identify uncultivable microbiota, while the pre-selection of primers and probes in closed-ended molecular methods biases microbial identification toward targeted species, excluding less-studied or ‘unexpected’ taxa [87]. Over the past decade, next-generation sequencing (NGS) technologies, which enable high-throughput DNA sequencing, have proven highly relevant for studying complex microbial communities. In endodontics, microbial identification has primarily relied on partial sequencing of the 16S ribosomal RNA (16S rRNA) gene. NGS profiling of root canal communities has revealed previously hidden microbial diversity, significantly expanding the catalogue of bacteria associated with these infections [88,89].

Scholars performed microbiome profiling to characterise the microbial communities associated with distinct infection origins and different anatomical regions of the root canal system. Sun et al. analysed the microbiome in patients with combined periodontal-endodontic lesions and found the relative abundances of *Clostridiales Incertae Sedis XI*, *Fusobacteriaceae*, *Fusobacterium*, *Parvimonas*, *Micrococcaceae* and *Rothia* were significantly increased in the pulp of retrograde pulpitis than non-necrotic inflamed pulp conditions [90]. In the infected root canal systems of extracted teeth with apical periodontitis, the apical samples were dominated by Proteobacteria whereas the coronal samples were enriched with Actinobacteria, and apical samples located statistically significantly more taxa than coronal samples, showing the complexity of root canal system infections [91]. Brito et al. reported that the microbial communities of endodontic infections at the apical portion and found 10 bacterial phyla, led by Bacteroidetes (51.2%) and Firmicutes (27.1%) and 94 genera represented primarily by Prevotella (17.9%) and Bacteroidaceae G-1 (14.3%) [92]. Ordinola-Zapata evaluated the root canal microbiome composition with primary and secondary root canal infections with whole-metagenome shotgun sequencing, and displayed that the taxonomic differences but the similar functional capability of the microbiomes was found in primary and secondary root canal infections of apical periodontitis patients [93]. These studies demonstrated that a pronounced bacterial diversity in the infected canal system, with a high interindividual variability and different microbiome compositions at the species/genus level were observed in the different infection type and sites [94]. Thus, in the following section, we present a comprehensive review of representative microorganisms in endodontic infections of different aetiologies.

### Microbes from coronal ingress

The infection pathway from dental crown is the most frequent and primary pathway for dental pulp infection. When the integrity of the enamel or cementum is destroyed, pathogens might provoke dental pulp through the exposed dentinal tubules in the oral cavity or directly infect the exposed dental pulp tissue, resulting in dental pulp infection. Subsequently, several typical pathogens derived from coronal ingress were introduced as follows (Table 1).

**Table 1.** The mainly infection passway, character and pathogenic mechanisms of representative microorganisms in endodontic infections.

The mainly infection passway	Microbes	Occurrence in progression of pulpitis	Pathogenic mechanisms	Positive effect on oral health
Coronal ingress	*Streptococcus mutans*	▪ Caries ▪ Initial stage of pulpitis	▪ Induce type 1 cytokine and activate CD8^+^ T cells [95] ▪ Induce pulp cell apoptosis [96] ▪ Escape from phagocytic killing by inducing NET formation [97] ▪ Bacteria cell-to-cell interactions [98]	
	*Lactobacillus* Species	▪ Deep dental caries ▪ Pulpitis, especially irreversible pulpitis	▪ Induce IL-6,10 and IFN-γ [99,100] ▪ Bacteria interactions by colonising and accumulating biofilms [101]	▪ anti-inflammatory effect ▪ Antibacterial and antibiofilm effect
	*Actinomyces* Species	▪ Caries ▪ Initiation of pulpitis	▪ Microial interactions [102] ▪ Develop sulphur granules and inhibit phagocytosis [103]	
	*Candida albicans*	▪ Caries ▪ Endodontic infections	▪ Bacteria interactions [104,105]	▪ stabilise oral ecosystem [106]
periodontal pocket	*Porphyromonas gingivalis*	▪ periodontitis ▪ retrograde pulpitis	▪ Inhibit mineralisation [14] ▪ evade immune responses [107]	
	*Capnocytophaga periodontitidis*	▪ gingivitis and periodontitis ▪ Pulp infection	▪ Remain to be investigated [108]	

#### Streptococcus mutans

*Streptococcus mutans*, a type of Gram-positive bacteria, is a major cariogenic organism due to its capacity to produce large amounts of glucans and acid [109]. This acid production surpasses the buffering capacity of saliva, allowing *S. mutans* to thrive and outcompete non-cariogenic commensal species in low pH environments [110]. The acidic environment it creates accelerates the process of enamel demineralisation [111–114], which eventually leads to dentin exposure and the onset of pulpitis.

*S. mutans* may have a major impact on the initial lesion and pulpal pathology. As one of the pioneers in pulp invasion to initiate endodontic infection [115], *S. mutans* elicits an extraordinary induction of type 1 cytokine and induces the preferential activation of CD8+ T cells, according with the aetiology of the CD8+ T-cell-dominant lesion in early pulpitis [95]. Additionally, LTA from *S. mutans* induced apoptosis of cultured pulp cells (mainly fibroblasts) in vitro, which could contribute to the initiation of and/or progression of pulpitis [96]. Despite triggering immune response, *S. mutans* is able to escape from phagocytic killing by inducing NET formation [97].

In addition, *S. mutans* can have cell-to-cell interactions with other plaque bacteria [98], attributing to the formation of dental plaque and a stronger virulence [101], which might help induce deep dental caries and even endodontic infections. These plaque-associated microorganisms might include *Lactobacillus casei* [101], *Candida albicans* [104,105] and *Veillonella parvula* [116].

#### *Lactobacillus* species

Like *S. mutans*, *Lactobacillus* are well known for their acidogenic and acidophilic properties [117], which gives them a comparative survival advantage. It is dominant in half of the deep dentinal caries [118], and its abundance continues to increase as the disease progresses, becoming prominent in the initial stages of polymicrobial infection of dental pulp [119]. Besides, *Lactobacillus* was reported to be the most frequent taxon in irreversible pulpitis [18].

*Lactobacillus* can invade intratubular and cause pulp inflammation, triggering various cytokine induction when the integrity of dentin was compromised. *Lactobacillus* might induce IL-10 and IFN-*γ* in inflamed pulp associated with deep caries [99], and might be involved in developing pulpitis through the stimulation of IL-6 production [100]. Conversely, *Lactobacillus* might have significant anti-inflammatory effect. It was suggested that probiotics (*L. rhamnosus* and *L. acidophilus*) had a significant effect on the severity of apical periodontitis in rats [120]. Besides, in spite of its low affinity to the tooth surface, *Lactobacillus* can make it up by colonising and accumulating biofilms during growth with other bacteria, including *S. mutans* and *Actinomyces spp.* [101], and accelerate the process from dental caries to pulp inflammation synergistically.

However, researches also suggested that *Lactobacillus* might have a positive effect on oral health. Antibacterial (pH-dependent) and antibiofilm activities of *Lactobacillus sp* against *S. mutans* can lead to reduction in microcolony formation and exopolysaccharide structural changes [120,121], which would be a promising strategy to prevent dental caries.

#### *Actinomyces* species

Actinomyces species are nonacid-fast, nonmotile and nonspore-forming Gram-positive rods. Mostly facultative anaerobes, and some are anaerobic, which explains their preference to low-oxygen environment [122]. Actinomyces may have a significant role in caries initiation, and occurs of high abundance in the earliest stage of caries [114]; however, as deep caries progressed toward pulpitis, its abundance begins to decline [119] and reaches approximately 9.4 to 15% of primary root canal infections [123–126].

The bacteria themselves exhibit limited pathogenic potential to directly induce pulp infection. Research have shown that very high numbers of Actinomyces cells are usually needed to form persistent infections [127]. Therefore, Actinomyces are always found in normal oral flora and induce pulpitis along with other bacteria [102]. A meta-analysis showed that, several bacteria genra were detected in association with bacteria of the genus Actinomyces, including Streptococcus, Propionibacterium and Eubacterium [128]. Actinomyces species is able to modulate PH and remove oxygen, thus creating a moderate and anaerobic milieu for other bacteria [129]. Additionally, it was reported that co-aggregation with A. naeslundii supports the growth of Streptococcus gordonii [130,131], which could and lead to synergistic pulp infection and periapical pathosis.

Strains of Actinomyces have been implicated in treatment failures [124]. The bacteria seem to be capable of evading host defences in a collective way by developing sulphur granules [103]. This might provide a resistance mechanism to host defences by inhibiting phagocytosis.

#### Candida albicans

As mentioned earlier, *C. albicans* can also have a role in caries pathogenesis and the fungus most frequently isolated from endodontic root canal infections [132]. *Candida albicans*, an opportunistic pathogen, is the most prevalent opportunistic fungal species in the oral cavity and colonises human mucosal surfaces commonly [133]. Its carriage rates vary widely: from 20 to 75% in the general population, 65 to 88% among individuals in acute and long-term care facilities, and up to 95% in people with HIV [134]. A meta-analysis showed that the overall prevalence of *Candida* spp. in root canal infections was 8.20%, among which *C. albicans* was the most frequently isolated species and was reported in approximately two-thirds of studies included [115].

The role of *C. albicans* remained to be investigated. Some studies have shown that *C. albicans* could be the partner with other cariogenic organism, since streptococci and other pathogens could adhere to *C. albicans*, supporting its colonisation and persistence [104,105]. Additionally, they produce lactate, which serves as a carbon source for yeast growth, while *C. albicans* lowers oxygen levels to those favoured by streptococci and supplies growth-promoting factors for the bacteria [110,135]. However, some scholars suggested that *C. albicans* has a positive role in oral ecosystem. *C. albicans* protected many bacteria to form biofilm, and healthy recolonisation process plays a crucial role in maintaining overall health. *C. albicans* in the oral cavity could contribute to the stability of the oral ecosystem [106]. C. albicans was reported to prevent caries by actively increasing pH preventing mineral loss with using an in vitro dual-species cariogenic oral biofilm model [136].

Despite being recognised by dental pulp and periradicular tissue cells that trigger immune responses, *C. albicans* evades host defences and induces cell death. It then attaches to tooth dentin, forms biofilms and invades dentinal tubules, making it resistant to intracanal disinfectants and endodontic treatments. Due to its insensitivity to common medications, *C. albicans* persists within biofilms and intratubular dentin, which has linked it to persistent or treatment-resistant root canal infections [132,137].

In addition, more metagenomics studies have also revealed associated other microorganisms, such as Epstein–Barr virus, *Rothia*, *Neisseria* and *Haemophilus* spp. and *Prevotella* spp. with caries and might be exist in dental pulp [138,139].

### Microbes from radicular retrogenic infection

#### Microbes from periodontal pocket

When a tooth is affected by severe periodontitis and the periodontal pocket extends to the apex, pathogens in the pocket might enter into the pulp chamber through the apical foramen or lateral canals, which is known as retrograde pulpitis clinically.

A wide range of periodontal pathogens have been implicated in this process. In particular, the relative abundance of *Fusobacterium*, *Parvimonas*, *Prevotella*, *Leptotrichia* and *Porphyromonas* was found to be significantly elevated in the pulp of retrograde pulpitis [90,140]. In addition, *Porphyromonas gingivalis* and *Capnocytophaga periodontitidis* represent two well-recognised species that can invade the pulp via the periodontal route, highlighting the intimate microbial connection between periodontitis and pulpal disease.

##### Porphyromonas gingivalis.

Porphyromonas gingivalis (*P. gingivalis*), a gram-negative anaerobe from *Bacteroides* genus, is widely recognised as a key pathogen in periodontal disease. Beyond its role in periodontitis, *P. gingivalis* can also infect the dental pulp via the periodontal pocket, thereby contributing significantly to retrograde pulpitis. It has been detected in both the pulp tissue and the adjacent deep periodontal pockets [11]. Moreover, *P. gingivalis* is frequently found in the apical portion of the root canal [141], and studies have shown that it is among the dominant bacteria present in persistent periapical lesions [142].

The bacterium and its major virulence factor, lipopolysaccharide (LPS), can affect dental pulp cells [143–146]. When hDPSCs are exposed to *P. gingivalis* LPS (Pg-LPS), the expression of mineralisation-associated genes such as *DSPP* and *OCD* is significantly downregulated [147]. Furthermore, *P. gingivalis* has been shown to evade immune responses within periodontal tissues [107].

Our studies demonstrated that *P. gingivalis* stimulation induces a range of matrix mineralisation-related changes in hDPSCs. Notably, we identified a distinct cluster of hDPSCs specifically responsive to *P. gingivalis*, characterised by high expression of *Thrombospondin 1 (THBS1)* and *Prostaglandin-Endoperoxide Synthase 2 (PTGS2)*. We designated these cells as *THBS1⁺* and *PTGS2⁺* hDPSCs [14].

##### Capnocytophaga periodontitidis.

*Capnocytophaga* is a genus of capnophilic, facultatively anaerobic, Gram-negative bacteria with gliding motility, classified under the family *Flavobacteriaceae* and the phylum *Bacteroidetes*. Species within this genus are considered commensals of the human oral cavity and are frequently detected in individuals with gingivitis [148] and periodontitis [149]. However, *Capnocytophaga* species may also function as opportunistic pathogens, particularly in individuals with compromised immune systems, and have been implicated in systemic infections [150]. The *Capnocytophaga periodontitidis* (*C. periodontitidis*) strain used in this study was isolated from dental plaque located in the deep periodontal pockets of patients with chronic periodontitis [151].

Our research demonstrated both homogeneous and heterogeneous responses of dental pulp stem cells (DPSCs) to infections by *C. periodontitidis* and *P. gingivalis*. Specifically, infection with *C. periodontitidis* promoted DPSC differentiation toward smooth muscle-related cell types, whereas *P. gingivalis* infection induced differentiation toward mineralisation-related lineages. A specific DPSCs subcluster uniquely responded to *C. periodontitidis* and aggregated under various periodontal infections, with differentially expressed genes linked to B-cell activation, muscle proliferation and ROS pathways [108].

#### Microbes from hematogenic infection

When the host is in a state of bacteraemia or sepsis, bacteria and toxins might irritate the pulp through the bloodstream, causing the inflammation of dental pulp and haematogenous pulpitis. It was theoretically possible based on ‘anachoresis’, which refers that blood-borne bacteria are easily attracted to and fixed in circumscribed areas of inflammation and being supported in dogs [152]. However, although oral bacteria are known to enter the bloodstream and cause systemic infections such as infective endocarditis, the reverse process – namely, haematogenous infection of the dental pulp – appears to be extremely rare and remains unconfirmed in routine clinical practice [153]. Therefore, the clinical relevance of haematogenous retrograde pulpitis remains questionable, and current evidence does not support it as a common or established pathway of pulp infection.

While categorising pulpitis-associated microbes by their routes of entry provides clinical relevance, another complementary perspective is to examine them at the phylogenetic level. Studies have shown that microorganisms from different phyla not only differ in their ecological niches but also trigger distinct host responses and inflammatory pathways. The following section therefore summarises the major microbial phyla involved in pulpitis and highlights their characteristic interactions with DPSCs.

### Microbial phyla and distinct host immune responses

From a host-defence perspective, responses to different microbial phyla involve coordinated actions of multiple cell subsets, including resident stromal cells (such as DPSCs and SCAPs) and immune effector cells (e.g. macrophages, neutrophils and lymphocytes), contributing to pathogen sensing, inflammatory amplification and tissue remodelling. Beyond the routes of entry, pulpitis-associated microbes can also be categorised by their phylogenetic distribution. Studies have shown that the majority of pathogens belong to four major phyla – Firmicutes, Bacteroidetes, Fusobacteria and Actinobacteria – each associated with distinct types of pulpitis and characteristic host responses [154,155]. These phyla differ not only in ecological niches but also in virulence strategies, thereby influencing both the progression of inflammation and the clinical manifestations of disease.

#### Gram-positive vs. gram-negative responses

Gram-positive and Gram-negative bacteria trigger overlapping but distinct immune responses in pulp tissues. Both groups induce IL-1 secretion, yet Gram-positive species elicit stronger IL-12 and TNF-*α* responses, largely due to lipoteichoic acid (LTA), a surface component that activates NF-κB and stimulates IL-6, IL-8 and TNF-*α* production in DPSCs, monocytes and leucocytes [156–160]. In contrast, Gram-negative bacteria preferentially induce IL-6, IL-1β and IL-8 in pulp cells [143,161–163]. Clinically, Gram-positive bacteria predominate in early carious lesions, whereas Gram-negative anaerobes increase in deeper or advanced lesions [28].

#### *Enterococcus faecalis* (Firmicutes)

*E. faecalis*, a Gram-positive facultative anaerobe, produces virulence factors such as LTA, gelatinase, hyaluronidase and cytolysin, which promote tissue destruction and immune modulation [164]. It induces IL-1β, TNF-*α*, TNF-*β* and IFN-*γ* production through innate immune activation involving macrophages and resident pulp cells, while concurrently suppressing SCAP proliferation and odontogenic differentiation through upregulation of VEGFA, RUNX2 and TBX3 [165,166].

#### *Porphyromonas* and *Prevotella* (Bacteroidetes)

Within the Bacteroidetes phylum, *Porphyromonas gingivalis* is a key retrograde pathogen that alters DPSC differentiation via TGF-β/SMAD, NF-κB and MAPK/ERK pathways. Its LPS reduces ALP activity and BSP expression while inducing stronger cytokine responses than *E. coli* LPS [14,167–169]. *Prevotella intermedia*, another member of this phylum, enhances NO and IL-1β release in macrophages, contributing to pulpal inflammation [170].

#### *Fusobacterium* (Fusobacteria)

*Fusobacterium nucleatumis* strongly associated with periodontal and systemic diseases, as well as pulp infections. It modulates SCAP gene expression by downregulating WDR5 and TBX2 while upregulating TBX3 and NFIL3, thereby impairing odontogenic differentiation. It also promotes IL-6, IL-8 and MCP-1 secretion and activates the STING pathway and autophagy, leading to IFN-*β* release [166,171,172].

#### *Actinomyces* (Actinobacteria)

*Actinomyces*, early colonisers of oral biofilms, interact with *Streptococcus* spp. to enhance IL-12p70 and IL-8 secretion, thereby facilitating endodontic infection [173]. Moreover, *A. actinomycetemcomitans* LPS is a potent inducer of IL-1α, IL-1β and bone resorption, even stronger than *P. gingivalis* LPS, highlighting its potential role in pulpal pathology [174].

Collectively, the evidence demonstrates that Firmicutes, Bacteroidetes, Fusobacteria and Actinobacteria employ unique pathogenic mechanisms to modulate host responses in the pulp, engaging distinct defence modules across immune cell subsets and stromal progenitor cells. Building upon these observations, we sought to examine whether such differences can be resolved at single-cell resolution by profiling DPSC responses to representative microbial species.

## Single-cell insights into DPSC responses to microbes

**Figure 4.**
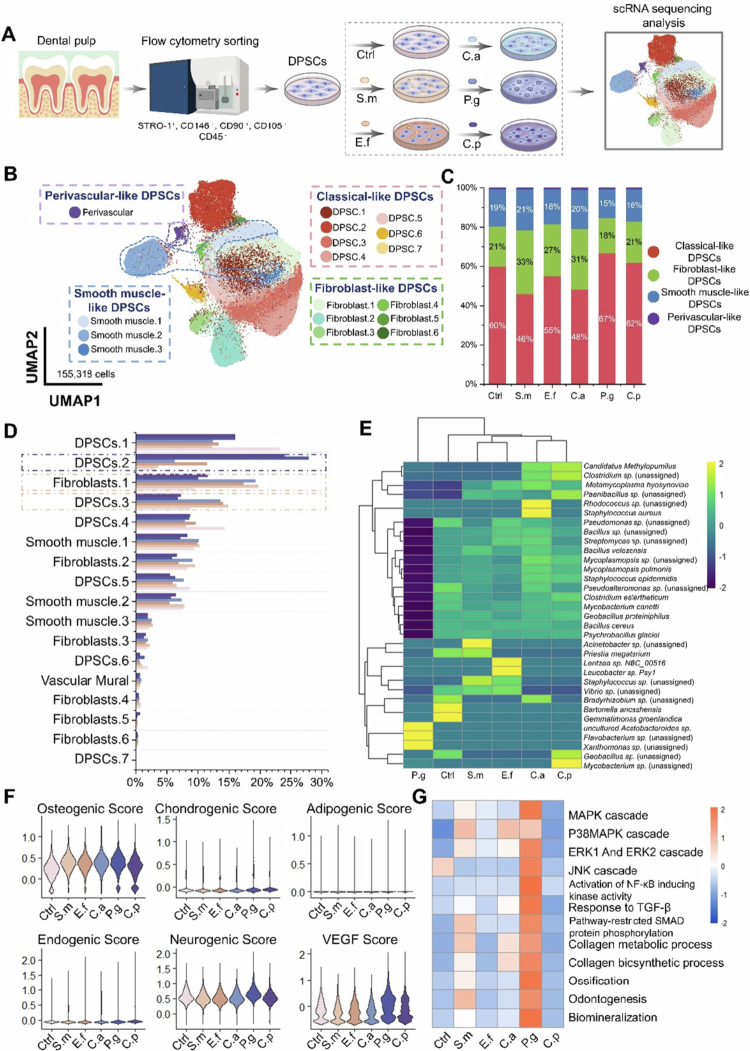
Single-cell RNA sequencing analysis of dental pulp stem cell responses to five representative pulpitis related oral microbes. (A). Diagram illustrating the experimental design strategy. (B). Distribution of 17 subclusters of DPSCs belonging to four major clusters, including classical-like DPSCs, fibroblast-like DPSCs, smooth muscle-like DPSCs, perivascular-like DPSCs, visualised in UMAP. (C). Bar graph of the cell proportion of four major cell types, divided by samples. (D). Bar graph of the cell proportion of 17 subcluster cell types, divided by samples. (E). Heatmaps showing the number of microbial taxa detected across different samples. (F). Violin plots showing the hDPSCs differentiation scores among the subtypes. (G). Heatmaps of enriched pathways among six samples.

Since DPSC was intimately associated with response after infection, our group explored the response of DPSCs to different microbes based on single-cell RNA sequencing, including *Streptococcus mutans* (*S. mutans*), *Enterococcus faecalis* (*E. faecalis*), *Candida albicans* (*C. albicans*), *Porphyromonas gingivalis* (*P. gingivalis*), *Capnocytophaga periodontitidis* (*C. periodontitidis*) [14,15]. In our review, we have collected six samples for horizontal comparison to explore the influence of dental pulp stem cells on the typical microbial species (Figure 4a).

A total of 155,318 cells were yielded after quality control. Seventeen subclusters were identified using unsupervised clustering and further annotated. As some subclusters has the similar cellular and gene expression pattern features, we integrated cell subclusters into four major clusters, including classical-like DPSCs, fibroblastic-like DPSCs, smooth muscle-like DPSCs and perivascular-like DPSCs (Figure 4b). The percentage of fibroblast-like DPSCs increased in S.m (33%), E.f (27%) and C.a (31%) samples, and the percentage of four major types of DPSCs were similar in P.g and C.p samples, suggesting that microbes from the same infection pathway may have similar effects on DPSCs (Figure 4c).

Several subclusters has distinctive effects when infected by microbial groups from dental crown (S. *mutans*, *E. faecalis* and *C*. *albicans*) or microbial groups from periodontal pocket (*P. gingivalis* and *C*. *periodontitidis*). The proportion of DPSC.2 increased obviously in groups from periodontal tissue, while the ratio of fibroblasts.1 and DPSCs.3 raised evidently in groups from dental crown (Figure 4d).

Then, we revealed cell-specific, species-specific bacterial burden and employed the ‘Single-cell Analysis of Host-Microbiome Interactions’ pipeline (SAHMI) to identify sparse bacterial reads from six samples. Interestingly, the infected microbial were not the main microbes found in DPSCs. More microbial types than we expected was analysed in six samples, demonstrating that more studies needed to be performed to investigate to understand the microbial status when infecting and entering into DPSCs. The infection pattern was different between groups from coronal ingress and those from the periodontal pocket (Figure 4e).

Then, we conducted the differentiation potential score analysis and computed the activity of classical pathway in GSEA database mentioned in previous studies. We found the similar clinical features was showed in the samples from the same infection sources. All infected samples got more osteogenic score than Ctrl samples, while more neurogenic and VEGF score was obtained in from P.g and C.p groups than S.m, E.f and C.a groups (Figure 4f). Interestingly, DPSCs from P.g samples showed the highest activity in the MAPK-related, NF-κB, TGF-β/SMAD, collagen metabolic and odontogenesis pathway and DPSCs from C.p samples has the lowest activity (Figure 4g).

**Figure 5.**
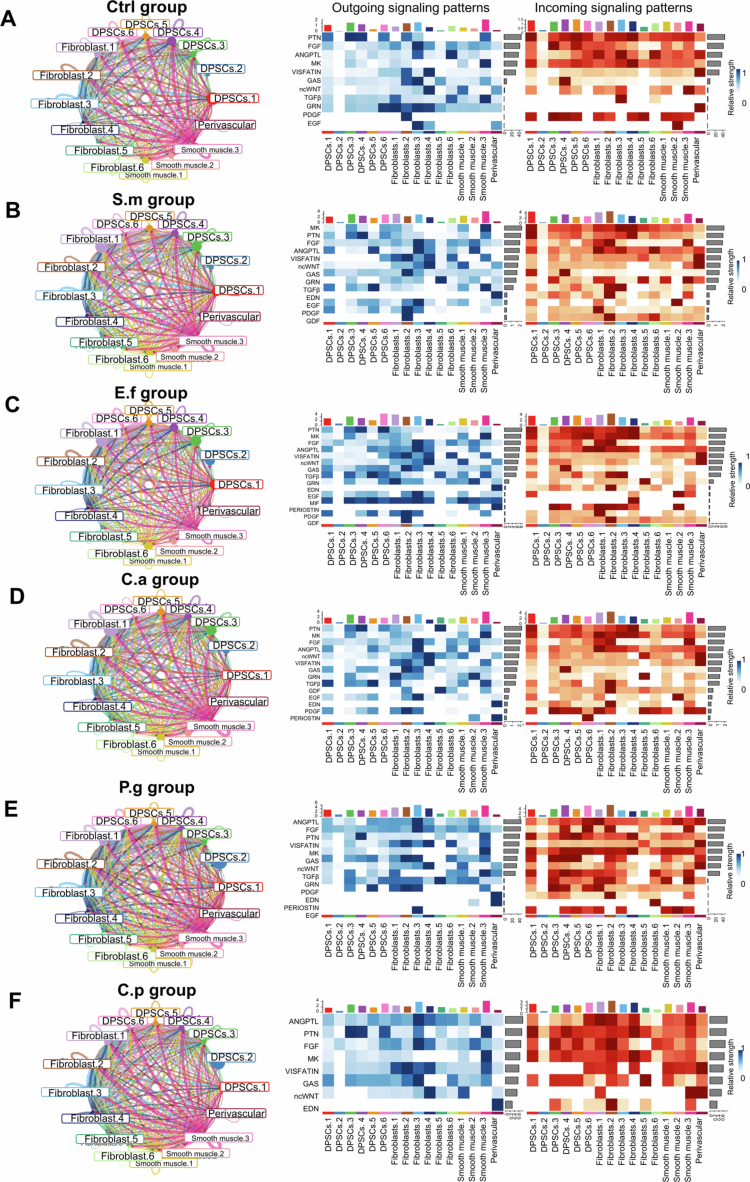
Cell–cell interactions among different subtypes. Circle plots showing the weight/strength of cell–cell interactions in left portion of Ctrl (A), S.m (B), E.f (C), C.a (D), P.g (E) and C.p (F) groups. The edge width is proportional to the indicated weight/strength of ligand–receptor pairs. Heatmaps (right) of signals contributing mostly to outgoing or incoming signalling of DPSCs clusters.

We further analysed the intercellular communication patterns and quantified input–output signalling strengths among different cell subclusters. We found that the pathway ranking profiles in the P.g and C.p groups were markedly different from those in the Ctrl, S.m, E.f and C.a groups. In the Ctrl group, signalling was dominated by PTN, MK, FGF, ANGPTL, VISFATIN, ncWNT, GAS and TGFβ, while the strength of other pathways was considerably lower. The S.m and C.a groups displayed patterns similar to Ctrl, but the relative intensity of PTN, MK and FGF signalling was reduced compared with other pathways. The E.f group also resembled Ctrl but included additional pathways such as MIF – which regulates cell migration, survival and angiogenesis – and Periostin, which is associated with tissue repair, fibrosis and inflammation, which is consistent with our previous findings. In contrast, in the P.g and C.p groups, ANGPTL (angiopoietin-like proteins, regulators of lipid metabolism, angiogenesis and inflammation) emerged as a top-ranked pathway, along with enhanced VISFATIN signalling (Figure 5). Collectively, these results highlight that different infection states shape distinct patterns of cell–cell interactions, and the observed alterations in signalling pathway strength may represent potential molecular indicators for predicting pulpal infection status.

More specific details were depicted in our studies previously published [14,15,108]. We conducted three comparative studies on every two samples, including comparison of different source of pathogen (E.f and P.g), different source of biological type (C.a and S.m) and the same periodontal pocket source of pathogen (C.p and P.g).

Compared E.f sample with P.g sample, DPSCs in P.g samples upregulated the expression of *THBS1*, *COL1A2*, *CRIM1* and *STC1* (matrix formation and mineralisation), and *HILPDA* and *PLIN2* (the cellular response to hypoxia), while DPSCs in E.f samples showing up-regulated expression associated with leucocyte chemotaxis (*CCL2*) and leucocyte chemotaxis (*ACTA2*). DPSCs differentiate into mineralisation-related cells when infected with *P. gingivalis* and into fibroblast-like cells when simulated by *E. faecalis* [14]. By comparing the C.a sample with the S.m sample, we found a subclusters, DPSC.7, which had a unique and specific change when infected with *C. albicans*. They would up-regulated MAPK/ERK1/2 and NF-κB pathway, suppress *DUSP1/5/6* expression, activate FOS, induce immune-related pathway and express cytokines, including *IL-6*, *CCL2* when stimulated by *C. albicans* [15]. Compared C.p sample with P.g sample, DPSCs were demonstrated to upregulate hypoxia and downregulate cell cycle-related pathway activity universally in the two infected samples. DPSCs infected with C.p. differentiated into smooth muscle-like cell lineages and DPSCs infected with P.g. differentiated into fibroblast (mineralised) lineages. A specific subcluster specially response to *C. periodontitidis* infection expressed genes associated with B cell activation, muscle cell proliferation and ROS-related pathway [108].

Together, our single-cell analyses reveal that distinct microbes elicit specific transcriptional and signalling programmes in DPSCs. Coronal ingress pathogens (*S. mutans, E. faecalis, C. albicans*) mainly promoted fibroblastic or immune-related responses, whereas periodontal pocket pathogens (*P. gingivalis, C. periodontitidis*) induced hypoxia, angiogenic or smooth muscle–like programmes. Intercellular communication patterns also shifted, with PTN-, FGF- and MK-dominated networks in coronal infections, and ANGPTL- and VISFATIN-driven signalling in periodontal infections. These pathogen-specific host responses highlight the heterogeneity of DPSC behaviour and suggest that molecular signatures derived from such interactions may serve as objective biomarkers for grading pulpal inflammation and guiding vital pulp therapy.

## Future perspectives

Accurate diagnosis of the pulp condition is crucial for the clinical success and prognosis of endodontic treatment. Evaluating the potential extent of inflammation and/or infection in the dental pulp is essential [175]. However, it is a lack of evidence to accurately assess the true condition of the pulp or to identify prognostic indicators that would enable a reliable preoperative estimation of the outcome of vital pulp therapy [13]. According to caries and pulpitis samples sequenced by 16S rDNA, the analyse of the microbiome was suggested to hold potential as a supplementary method for diagnosing pulpitis and future pulpitis onsets for samples clinically perceived as healthy based on the dental caries microbiome [18,139]. Nevertheless, methods for assessing pulp status remain underexplored and require further refinement.

Our findings, together with previous reports, suggest that different microorganisms can elicit distinct and specific host responses in dental pulp stem cells (DPSCs). These pathogen-specific cellular and molecular signatures could serve as objective ‘cellular or molecular maps’ for grading inflammation severity and guiding treatment decisions. We therefore advocate for the establishment of a comprehensive single-cell response atlas of DPSCs to representative pulp pathogens, which could form the foundation for developing biomarker-driven diagnostic models to support VPT decision-making.

Despite decades of research, significant uncertainty persists regarding the immunological processes and structural changes within the dental pulp at both the tissue and cellular levels. While the immune cell composition in healthy and diseased pulp has been broadly characterised, the specific functions of immune cell subsets and their interactions with DPSCs under infection by different pathogens remain poorly understood. Moreover, the inflammatory response is inherently complex, with spatial and temporal heterogeneity that is still incompletely described. In addition, single-cell–based models are inherently variable due to experimental conditions, microbial exposure and donor heterogeneity, which may affect the interpretation of transcriptomic signatures. Translating these findings into clinical diagnostics remains challenging and will require validation in larger cohorts and standardised, chairside-applicable biomarker assays.

Looking ahead, recent methodological advances such as spatial omics offer powerful tools to resolve the three-dimensional organisation of immune and stromal interactions within the inflamed pulp. Coupled with targeted biomarker discovery and artificial intelligence–based analytical frameworks, these approaches hold promise for transforming pulpitis diagnosis from a largely subjective clinical judgement into an evidence-based, precision-guided process. Such integration of microbiology, single-cell biology and computational modelling could ultimately enable personalised, minimally invasive endodontic care that optimises pulp preservation [29].

## Conclusion

In conclusion, elucidating the microbial origins and pathogenic mechanisms of pulpitis is fundamental to advancing both diagnostic precision and therapeutic efficacy. In this review, we summarise the diverse infection routes, microbial taxa and virulence characteristics associated with pulpitis and discuss their corresponding host immune and repair responses. Our single-cell analysis of dental pulp stem cell (DPSC) responses to representative pathogens demonstrated that different microorganisms can elicit distinct cellular and molecular programmes, reflecting variations in infection mechanisms and potential disease severity. These pathogen-specific signatures hold promise as objective biomarkers for grading pulpal inflammation and guiding clinical decision-making in vital pulp therapy. Looking forward, integrating microbial profiling with single-cell atlases, spatial omics and AI-driven analytical models may enable the development of personalised, biomarker-based strategies that optimise pulp preservation and improve regenerative outcomes in endodontic care.

## Data Availability

Data sharing not applicable to this article as no datasets were generated or analysed during the current study.
